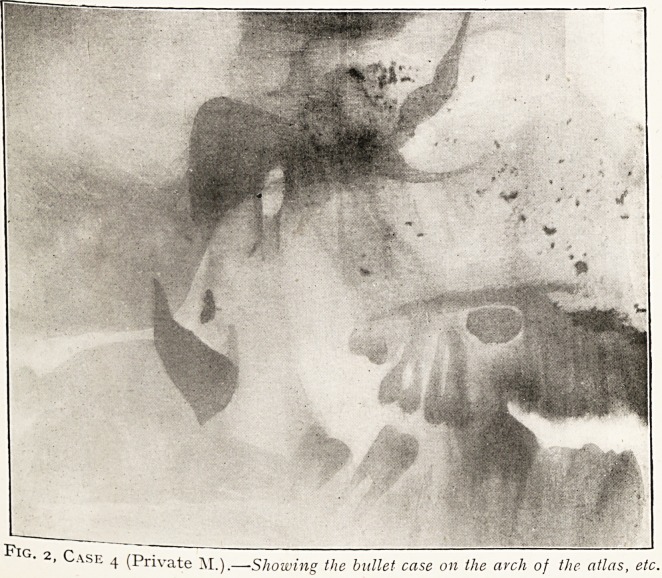# Experiences of an Ear, Nose and Throat Specialist at One of the Bases

**Published:** 1916-07

**Authors:** J.P.I. Harty


### EXPERIENCES OF AN EAR, NOSE AND THROAT SPECIALIST AT ONE OF THE BASES.

BY

CAPTAIN J. P. I. HARTY, R.A.M.C.T., F.R.C.S.

---

OUR work here is arranged on practically the same principles as those of a civil clinic. We have the out-patient system, which was sanctioned by the D.D.M.S. owing to the number of cases attending for consultation from the various hospitals, while those from the base depôts, etc., receive daily treatment in addition where necessary.

We have a special hut set apart for the out-patient department. This is divided into a waiting-room capable of holding 80 to 100, and an examination room screened off so that two officers can work there, and also a dark room for trans-illumination.

Captain Just came to assist me about a month after my arrival, and even with his help the work is as much as we can accomplish.

Our largest number of new cases in one day was 52, and the number attending for daily treatment at one time averaged 120. We found it necessary to reserve one day a week for operations, seeing no new cases on that day, the daily attendances only receiving treatment.

The appended statistics are compiled for the period January 1st to May 15th, 1916, inclusive.

1. Full list of cases, their diagnosis and disposal.

2. List of operations.

One great advantage which we have is that the treatment is administered daily by skilled hands, and not left to the patients themselves. This is especially valuable in the treatment of one of the biggest leakages of the army, viz. middle-ear suppuration, and we have found that in suitable cases these dry up soon, and we are able to return to duty a large number of men who were previously being sent to England.

We have been very lucky in obtaining the assistance of a most capable sister, and one who takes an interest in the work.

I was fortunate enough to obtain printed cards with diagrams modelled on the same principle as those used at the Bristol Royal Infirmary, and these have minimised our work, and made the records of the cases complete and concise. Comparing the statistics with those of a civilian clinic the differences are not great.

We have an almost complete absence of malignant cases and a very small percentage of tuberculous. This discrepancy is only to be expected on account of the age limit and the chest examination on enlistment. Sinusitis is a little less frequent, and this can be accounted for by the fact that we are working with males. I can say without hesitation that it is not a case of missing them, as in every case a complete examination of the nose and nasopharynx is made, and on any indication a sinus exploration is undertaken and skiagrams consulted in the case of suspected frontal sinusitis.

The war casualties which appear amongst our statistics comprise the following :—

A. Gunshot wounds of the face, jaws and ears.

B. Results of shell concussion on the middle internal and external ears.

C. The neurasthenic complication functional aphonia, which is fairly common.

We have a great preponderance of cases of middle-ear suppuration, possibly due to the difficulty in eliminating such cases at the medical examination for enlistment, as fully 50 per cent. of those examined with this complaint have had it when they joined.

It may be of interest to summarise our experience of the results of shell concussion on the ears. They may be divided into the following groups :—

1. *Otitis externa without implication of the middle ear.*

The percentage of these cases is hard to estimate, but undoubtedly some are due to this cause.

2. *Rupture of the membrana tympani.*

Of these three sub-groups may be mentioned :—

(*a*) Perforation without infection of the middle ear.

(*b*) Perforation with infection.

(*c*) Perforation complicated with nerve deafness.

With regard to the rupture, we have found that practically the pathognomic form is the kidney shape, with the apex of the handle of the malleus at the hilum.

In one case I have seen two perforations, the smaller one being situated in the postero-superior quadrant, and rather indicating it to have been due to direct violence, some small foreign body having been driven through as a result of the explosion. The larger one was of the type described above.

Infection is, as would be expected, fairly common owing to the fact that particles of dirt are carried in either from outside or from the meatus, which under war conditions is anything but aseptic. Nerve deafness is uncommon with perforation, the simple explanation being that the concussing force is taken by the membrane and its rupture saves the internal ear in most cases.

It is possible that in the perforation cases there is a rapid reflex action of the tensor tympani tightening the membrane, and the brunt of the concussion is borne in this way. It has been, however, our experience that nerve deafness without perforation is often associated with an atrophic condition of the membrana tympani.

3. *Nerve deafness.*

This has been classified into two sub-groups :—

(*a*) Auditory concussion.

(*b*) Labyrinthine hemorrhage.

We have not had the opportunity of investigating these, as the main differences seem to be that the former will at all events partially recover after some months, while the latter is permanent.

4. *Nerve deafness superimposed upon ears, either in a state of chronic suppuration, or with dried-up perforations, the results of earlier suppuration.*

We have had a fair number of these, and in the absence of the protecting influence of the membrane the internal ear naturally suffers.

5. *Subluxation of the stapes.*

Two instances of the above have come under our notice where there was a markedly indrawn membrane on one side with a negative Rinne, and a normal bone conduction combined with a clear history of concussion. A single inflation by means of the Eustachian catheter cured these, a positive Rinne and perfect hearing resulting.

Subjoined here are the statistics of these cases :—

*Shell Concussion Effects on the Middle and Internal Ears.*

| | |
|---|---|
| Nerve deafness .. .. .. .. .. .. .. | 31 |
| Concussion perforation *without* infection .. | 3 |
| Concussion perforation *with* infection .. .. | 15 |
| Concussion perforation *without* infection combined with nerve deafness[1] .. .. .. .. | 5 |

[1] Subluxation on the left side and perforation with nerve deafness on the right included in the above.

| | |
|---|---|
| Concussion perforation *with* infection combined with nerve deafness .. .. .. .. .. | 8 |
| Concussion nerve deafness superimposed on old chronic suppurative otitis media .. .. .. | 10 |
| Subluxation of the stapes .. .. .. .. .. | 1 |
| | 83 |

Included in the above are two rather interesting cases illustrating these types.

Two men came up together. On examining the first, he was found to have the characteristic perforations in both ears, non-infected and without any nerve involvement. His history was that a trench mortar had burst near him five days previously. The second man was standing near the first, but he was buried by the explosion, and had to be dug out. Just when he was free another trench mortar exploded and buried him a second time, and sad to relate on extricating himself a third burst quite close. This man on the right side had a characteristic concussion perforation, evidently produced by the first explosion, and a pronounced nerve deafness superimposed by the later explosions. On his left side he had escaped with a negligible amount of nerve involvement and a subluxation of the stapes cured by inflation.

With regard to the sinusitis cases, our explorations of the maxillary and sphenoidal sinuses have been made by the suction syringe method of Watson-Williams, which has proved reliable. The actual number of explorations made has been :—

| *Sinus.* | *Positive Result.* | *Negative Result.* |
|---|---|---|
| 75 Maxillary antra .. .. | 32 | 43 |
| 13 Sphenoidal .. .. .. | 5 | 8 |

Sinus operations have been performed where thought advisable, and if necessary the cases transferred to England for a continuance of treatment, but a fair number have been returned to duty, cured, direct from here. The intranasal operations have been performed in all cases with one exception, a frontal sinus seen in the late stage where the anterior wall had necrosed and osteomyelitis of the frontal bone had already started. This was treated by the external method with obliteration of the sinus, but unfortunately proved fatal owing to rapid extension of the osteomyelitis with infection of the meninges, etc.

The endonasal method of Watson-Williams has been used for entering the frontal sinus, and our results have been very satisfactory, enabling us to return some cases to duty after a short period of daily washings out.

The statistics show a large number of operations performed for nasal obstruction, especially those due to septal deflections. In this war clear nasal passages are absolutely essential, as when a man is wearing his gas helmet he is solely dependent on his nasal respiration. The history most frequently is that the man is unable to wear the gas helmet, or when he has it on that he is incapable of performing any active movement.

I am including short notes on a few cases which I think might be of interest.

**Case 1.**—Sergt. G. W. This man complained of pain in the left ear, especially after being on the rifle range. On examination he was found to have a small scar just in front of the left tragus, which he said was due to the removal of an accessory auricle in childhood. Otoscopy revealed a small horn-like ecchondrosis growing from the anterior meatal wall which curved inwards and almost touched the membrane. A patch of hemorrhage was seen on the membrane at what must have been the point of contact during any excessive excursion of the conducting mechanism.

The ecchondrosis was successfully removed by the meatal route and the man returned to duty.

**Case 2.**—Lance-Corpl. M. Admitted here May 1st. On April 25th a trench mortar burst close to him, and a fragment was driven through the left side of the bridge of his nose, fracturing

FIG. 1, CASE 2.—*Lateral skiagram. Nose to right.*

FIG. 2, CASE 2.—*Antero-posterior skiagram showing the shrapnel fragment in the right sphenoido-pituitary region.*

the left nasal bone and making a distinct window. His mental condition was good, but he was suffering from a ptosis of the right eye, and on more careful examination he was found to have right-sided complete ophthalmoplegia with anæsthesia over the distribution of the frontal nerve. Skiagram, prints of which are attached, showed the irregular shaped foreign body to be situated in the region of the pituitary fossa. On May 4th, under general anæsthesia and using Killian's long speculum, the missile was found to have tracked across the nose perforating the upper portion of the septum; it then passed through the right sphenoidal sinus, and the foreign body could be seen through a hole in the posterior wall of the sinus. Attempts to remove it with forceps and also with the magnet were unsuccessful. May 5th another attempt was made, and this time the aperture in the posterior sinus wall was carefully enlarged, and a right-angle seeker passed so as to curve behind the foreign body, which was thus dislodged, and after some difficulty, owing to the depth, seized with a pair of nasal packing forceps and extracted. The dura mater on careful inspection seemed to be intact. The cavity was swabbed out with collosol argentum, and subsequently spraying with the same fluid frequently employed. The patient, although never feeling very bad, said he was much better, and a slight headache he had previously disappeared.

Captain Pringle examined the eye condition for me, and agreed with the ophthalmoplegia diagnosis. The fracture had evidently run into the sphenoidal fissure and implicated all the nerves. He still had at this later date, May 22nd, almost complete loss of all ocular movements.

Paralysis of accommodation with a dilated pupil.

Ptosis.

The vision is not impaired beyond what can be accounted for by the loss of accommodation.

Visual fields full.

Discs normal.

Vessels normal.

The slight movement of the globe is due to the external rectus. Whether this indicates the initial stage of recovery starting in the sixth nerve time alone will show, but Captain Pringle thinks the prognosis is bad.

The patient has been able to get about for some days, and on May 22nd I freshened up the edges and brought them together, closing the entrance wound.

**Case 3.**—Private B. Shrapnel ball in right maxillary antrum. This man was seen on January 28th. He was wounded on January 10th, the ball entering the right orbit. Enucleation of the globe was performed on January 11th at the C.C.S. Admitted here February 7th. The skiagram shows clearly the size and position of the ball. Rhinoscopy revealed a perfectly clean and roomy right nasal cavity, but no sign of the foreign body.

After careful localisation by stereoscopic radiology, operation was undertaken on February 13th. Under general anæsthesia an intranasal entrance to the antrum was made, and a little finger introduced could feel the foreign body partially fixed in the roof. It was easily dislodged, but owing to its size extraction was difficult, so the patient was taken into the X-ray room, and a forceps having been passed in, with the help of the screen it was an easy matter to seize the ball and extract it, although the combined girth necessitated a strong pull when passing through the anterior naris. Recovery was very rapid, no temperature, and the antrum perfectly clean after a few washings out. The patient was sent to England on February 18th. Skiagram print attached.

**Case 4.**—Private M. This case, unfortunately, had a fatal termination. Admitted here April 30th.

*History.*—On April 27th he was struck by a rifle bullet in the left infraorbital region. His eye was removed at the C.C.S. He came down with a tube inserted along the track of the bullet. The tube was taken out and a glycerine and saline

FIG. 1, CASE 3.—*Shrapnel ball in the right maxillary antrum.*

FIG. 2, CASE 4 (Private M.).—*Showing the bullet case on the arch of the atlas, etc.*

dressing applied. The attached skiagram speaks for itself. The projectile had travelled almost horizontally backwards and to the right, and all the small shadows represent portions of the interior of the bullet, the main shadow resting on the right side of the arch of the atlas being the distorted case.

May 1st. Operation under a general anæsthetic. An attempt was made to remove the foreign body through the mouth via the isthmus pharyngei; this was unsuccessful. Right and left intranasal antral operations were then performed. The antra were washed out that evening, and subsequently twice daily. The mouth was sprayed frequently with collosol argentum, and the nasopharynx on the right side where the finger could feel comminution in the pterygoid region was also washed out twice daily with a solution of collosol argentum in normal saline (1 in 3) by means of a Eustachian Catheter.

The patient's condition rapidly improved, as the temperature chart shows, and on May 7th the fluid on washing out ceased to emerge from the infraorbital wound, which was quite healed on May 10th. On May 11th, under general anæsthesia, an incision was made behind the sternomastoid which was retracted forwards with the vessels, and then by working upwards the foreign body, found to be a distorted bullet case, was eventually removed with great difficulty. Major Tabuteau assisted me, and eventually was responsible for the extraction.

Unfortunately, infection of the most virulent type ensued, spreading amongst the deep tissues and under the prevertebral fascia, with a resulting mediastinitis and death on May 15th.

TOTAL LIST OF CASES.

| | Total List. | Disposal. | | | | | |
|---|---|---|---|---|---|---|---|
| | | Returned to duty. | Sent England. | Fit for Base duty. | Attending at the time. | In Wards. | Deaths. |
| NOSE. | | | | | | | |
| Sinusitis.—Frontal .. .. .. | 4 | 2 | 1 | — | — | — | 1 |
| Maxillary .. .. | 28 | 5 | 17 | — | — | 6 | — |
| Sphenoidal .. .. | 2 | — | 1 | — | — | — | 1 |
| Frontal and Maxillary .. .. | 4 | — | 3 | — | — | 1 | — |
| Pansinusitis .. .. | 6 | — | 6 | — | — | — | — |
| Polypi and Ethmoiditis .. .. | 9 | 4 | 5 | — | — | — | — |
| Deflected Septum .. .. .. | 88 | 62 | 15 | 1 | 3 | 7 | — |
| Hypertrophic Rhinitis .. .. | 57 | 45 | 2 | — | 8 | 2 | — |
| Atrophic Rhinitis .. .. .. | 12 | — | 9 | 2 | 1 | — | — |
| Congenital Syphilis .. .. | 2 | — | 2 | — | — | — | — |

TOTAL LIST OF CASES (*continued.*)

| | Total List. | Disposal. | | | | | |
|---|---|---|---|---|---|---|---|
| | | Returned to duty. | Sent England. | Fit for Base duty. | Attending at the time. | In Wards. | Deaths. |
| LARYNX AND PHARYNX. | | | | | | | |
| Pharyngitis .. .. .. .. | 20 | 15 | — | — | 5 | — | — |
| Acute Laryngitis .. .. .. | 93 | 69 | 16 | — | 7 | 1 | — |
| Chronic Laryngitis Papilloma | 2 | — | 2 | — | — | — | — |
| Tuberculous | 2 | — | 2 | — | — | — | — |
| Specific .. | 2 | — | 2 | — | — | — | — |
| Simple .. | 12 | 5 | 6 | 1 | — | — | — |
| Post-Cricorid Malignant Growth | 1 | — | 1 | — | — | — | — |
| Neurosis .. .. .. .. .. .. | 2 | 2 | — | — | — | — | — |
| Functional Aphonia .. .. | 21 | 11 | 8 | — | 2 | — | — |
| Tonsillitis and T.'s and A.'s .. | 101 | 77 | 3 | — | 7 | 14 | — |
| Syphilis of Nose, Pharynx and Larynx .. .. .. .. .. .. | 6 | — | 6 | — | — | — | — |
| EARS. | | | | | | | |
| Cerumen .. .. .. .. .. .. | 53 | 49 | — | — | 4 | — | — |
| Otitis Externa .. .. .. | 122 | 88 | 7 | — | 22 | 5 | — |
| Chronic Catarrhal Otitis Media | 253 | 161 | 26 | 38 | 24 | 4 | — |
| Acute Suppurative Otitis Media | 48 | 29 | 10 | — | 6 | 3 | — |
| Acute Catarrhal Otitis Media | 30 | 21 | — | — | 9 | — | — |
| Chronic Suppurative Otitis Media .. .. .. .. .. .. | 689 | 231 | 314 | 50 | 78 | 13 | 3 |
| Old Radical Mastoid Cases .. | 18 | 6 | 9 | 3 | — | — | — |
| Otosclerosis .. .. .. .. | 9 | — | 9 | — | — | — | — |
| Nerve Deafness .. .. .. | 12 | — | 7 | 5 | — | — | — |
| Labyrinthine Deafness post Cerebro-Spinal Fever .. | 1 | — | 1 | — | — | — | — |
| G.S.W. | | | | | | | |
| Concussion, Nerve Deafness, Perforations, etc. .. .. | 86 | 23 | 46 | 10 | 7 | — | — |
| Stenosed External Auditory Meati .. .. .. .. .. .. | 2 | — | 2 | — | — | — | — |
| Maxillary Antra, etc. .. .. | 18 | 5 | 6 | — | 2 | 4 | 1 |
| Unclassified .. .. .. .. .. .. | 75 | 64 | 8 | — | 3 | — | — |
| | 1890 | 974 | 552 | 110 | 188 | 60 | 6 |

OPERATIONS.

| | |
|---|---|
| Tonsil enucleations and adenoid removal .. | 74 |
| Minor operations .. .. .. .. .. .. | 62 |
| Submucous resections .. .. .. .. .. | 64 |
| Intranasal maxillary antral operations .. | 16 |
| Intranasal frontal sinus operations .. .. | 7 |
| External frontal nasal operations .. .. | 1 |
| Removal of ecchondrosis of external auditory meatus .. .. .. .. .. .. .. .. | 1 |
| Removal of foreign body from pituitary fossa (two operations) .. .. .. .. .. .. | 2 |
| Mastoid incomplete operation .. .. .. | 4 |
| Mastoid incomplete operation combined with obliteration of lateral sinus, etc. .. .. | 1 |
| Operation for stenosed external auditory meatus .. .. .. .. .. .. .. .. | 2 |
| | 234 |

## Figures and Tables

**Fig. 1, Case 2. f1:**
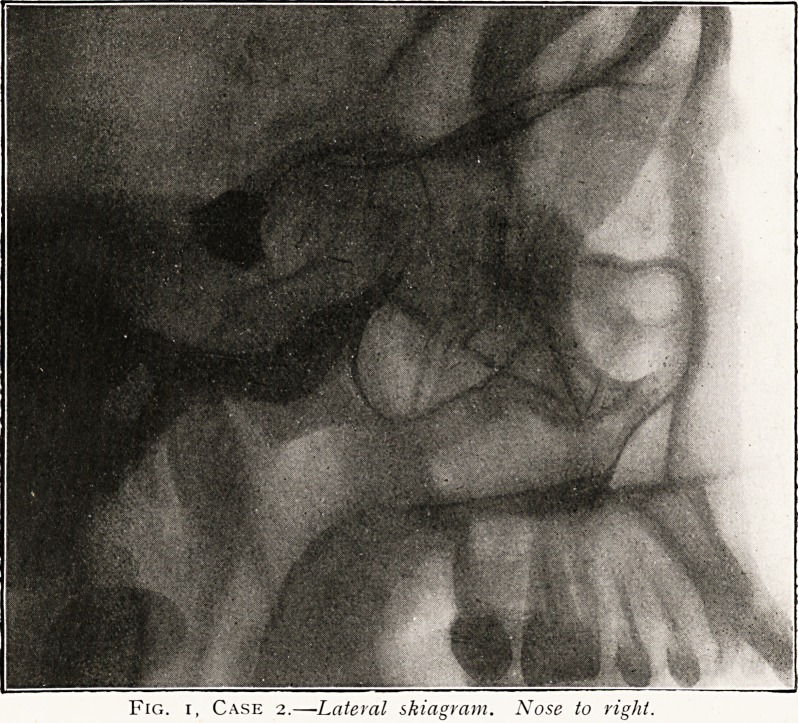


**Fig. 2, Case 2. f2:**
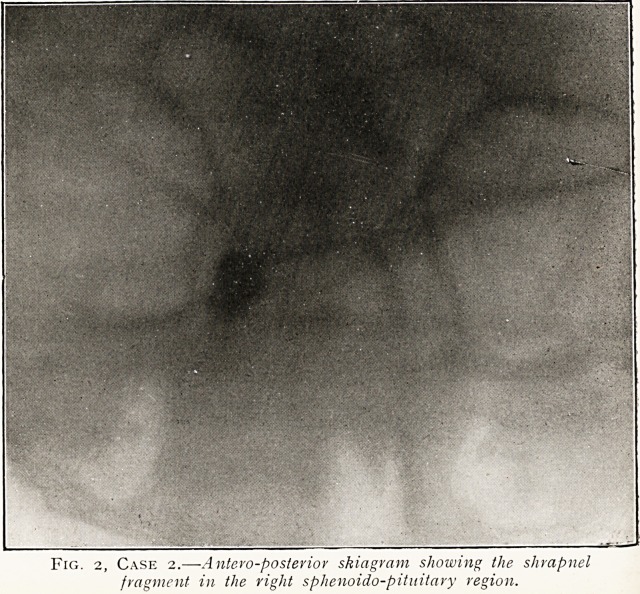


**Figure f3:**
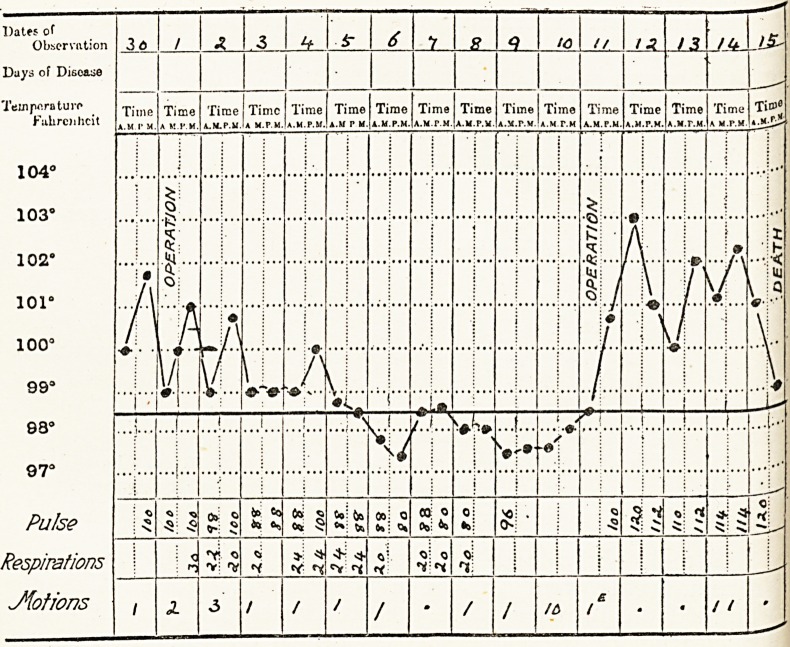


**Fig. 1, Case 3. f4:**
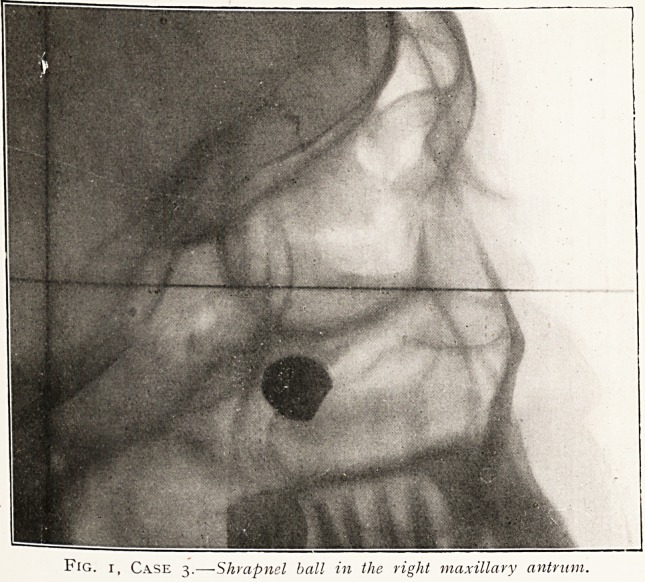


**Fig. 2, Case 4 (Private M.). f5:**